# Expectations and experiences with physician care among patients receiving post-acute care in US skilled nursing facilities

**DOI:** 10.1186/s12877-020-01869-1

**Published:** 2020-11-10

**Authors:** Kira L. Ryskina, Kierra A. Foley, Jason H. Karlawish, Joshua D. Uy, Briana Lott, Erica Goldberg, Nancy A. Hodgson

**Affiliations:** 1 Division of General Internal Medicine, Perelman School of Medicine of the University of Pennsylvania, Philadelphia, PA USA; 2 Leonard Davis Institute of Health Economics, University of Pennsylvania, Philadelphia, PA USA; 3 Department of Biobehavioral Health Science, University of Pennsylvania School of Nursing, Philadelphia, PA USA; 4 Division of Geriatrics, Perelman School of Medicine of the University of Pennsylvania, Philadelphia, PA USA; 5 VA Greater Los Angeles Healthcare System, West Los Angeles, CA USA; 6 Department of Emergency Medicine, Perelman School of Medicine of the University of Pennsylvania, Philadelphia, PA USA

**Keywords:** Caregivers, Patient satisfaction, Post-acute care

## Abstract

**Background:**

In the US, post-acute care in skilled nursing facilities (SNFs) is common and outcomes vary greatly across facilities. Little is known about the expectations of patients and their caregivers about physician care during the hospital to SNF transition. Our objectives were to (1) describe the experiences and expectations of patients and their caregivers with SNF physicians in SNFs, and (2) identify patterns that differed between patients with vs. without cognitive impairment.

**Methods:**

This qualitative study used grounded theory approach to analyze data collected from semi-structured interviews at five SNFs in January–August 2018. Patients admitted for short-term SNF care 5–10 days prior were eligible to participate. Thematic analysis was performed to detect recurrent themes with a focus on modifiable aspects of physician care. Analysis was stratified by patient cognitive impairment (measured by the Montreal Cognitive Assessment at the time of the interview).

**Results:**

Fifty patients and six caregivers were interviewed. Major themes were: (1) patients had poor awareness of the physician in charge of their care; (2) they were dissatisfied with the frequency of interaction with the physician; and (3) participants valued the perception of receiving individualized care from the physician. Less cognitively impaired patients were more concerned about limited interactions with the physicians and were more likely to report attempts to seek out the physician.

**Conclusion:**

Patient and caregiver expectations of SNF physicians were not well aligned with their experiences. SNFs aiming to improve satisfaction with care may focus efforts in this area, such as facilitating frequent communication between physicians, patients and caregivers.

**Supplementary Information:**

**Supplementary information** accompanies this paper at 10.1186/s12877-020-01869-1.

## Background

One quarter of the 1.5 million patients discharged annually from US hospitals to post-acute care (typically short-stay) in skilled nursing facilities (SNFs) are re-hospitalized or die within 30 days [1]. In the US, post-acute care in SNFs typically involves short-term rehabilitation services, occupational therapy and/or skilled nursing care aimed to improve the patient’s functional status and address any skilled nursing needs prior to transition back to the community. Patients with Alzheimer’s disease or related dementias are at an increased risk of rehospitalization from SNFs [2]. While physicians (and advanced practitioners such as nurse practitioners and physician assistants) are considered integral to improving care coordination and transitions in care [3, 4], little is known about their role in the outcomes of patients who transition from hospital to post-acute care in SNFs. Because patients with cognitive impairment are particularly reliant on others (e.g., their physicians and caregivers) to communicate medical information and treatment preferences, their experience and access to physicians after transfer to a SNF may provide insight into drivers of high hospital readmission rates and adverse events in this high-needs population.

Patient expectations and perceptions of the physicians’ care in SNFs are poorly understood. Patient-perceived deficiencies in SNF care include poor communication and care coordination between physicians and other medical staff as well as with patients and caregivers [5, 6]. However, prior research examining patient experiences in these facilities did not focus on patients with dementia who have complex needs and unique concerns. Furthermore, recent studies found that physician practice in SNFs is rapidly changing. Some physicians and nurse practitioners are increasingly focusing their practice exclusively on treating patients in these facilities (colloquially known as “SNFists”) [7–9]. Although one study using Medicare data found that patients under the care of SNFists have slightly better clinical outcomes [10], it did not evaluate patient perceptions of their care.

Strong physician-patient alliance is associated with improved outcomes across a range of medical conditions [11–13]. Awareness and understanding of patient expectations is key to building patient alliance with the physician, coordinating the care and improving the experience with and outcomes of SNF care. This may be particularly true for patients with dementia who more frequently rely on others (including caregivers and physicians) to advocate in healthcare settings. In the United States, a marked heterogeneity in physician practice arrangements within SNFs has been well documented, ranging from facilities staffed full-time by physicians who focus their practice on SNF-based care to facilities with open-staff models where generalist physicians from the community alternate seeing patients admitted to the facility [14, 15]. Little is known about patient and caregiver expectations and experiences of physician care across the spectrum of SNF-physician practice arrangements. Thus, there is an urgent need to evaluate patient and caregiver experiences and expectations of physician care in SNFs within the context of SNF-physician practice models. Furthermore, as the COVID-19 pandemic exposed significant vulnerabilities of SNFs as a provider of post-acute and long-term care for older adults, impending redesign of post-acute care delivery should be informed by empirical evidence of patient and caregiver preferences for care.

This study describes patient and caregiver expectations and experiences with SNF physician care among patients with and without cognitive impairment receiving post-acute care in these facilities. The primary aims of this study are: 1) to describe patient and caregiver expectations and experiences with SNF physicians among patients receiving post-acute care in these facilities (short-stay); and 2) to elicit thematic differences in patient and caregiver experiences among patients with and without cognitive impairment. In a secondary aim, we also evaluated thematic differences in patient and caregiver experiences among patients in SNFs where physicians focused their practice exclusively in SNFs (i.e., SNFists) vs. SNFs that employed traditional physician practice models (where physicians follow their patients across care settings including clinic, hospital, SNF, etc). We hypothesized that patients in SNFs that predominantly employ SNFists would have better experiences with physician care compared to the patients in SNFs that use physicians who do not focus their practice on SNF-based care.

### Conceptual model

Our conceptual model was based on the nursing home Medical Staff Involvement Model [6], which applied the Donabedian “structure-process-outcomes” framework [16] to describe the relationship between staffing and outcomes in the nursing home (or SNF). It was further developed using the influential framework of medical staff organization in hospitals by Roemer & Friedman [17] that identified job commitment of physicians as a key dimension influencing hospital performance. Based on qualitative studies of patients and family members, as well as administrators and other staff in SNFs [18–22], the Medical Staff Involvement Model links physician practice characteristics to care coordination processes that influence patient outcomes. We theorize that the degree to which SNF physicians focus on SNF-based practice and the practice strategies these physicians use to deliver care to this high-risk patient population are key to physician effectiveness in processes of care such as communication with patients and their caregivers, care coordination with the discharging hospital and external providers, and cross-coverage of acute patient needs. Since these processes are identified as particularly important to patients in SNFs and their caregivers in prior research that focused on end-of-life care [22–24], we expect that short-stay SNF patients and their caregivers will have expectations of their physicians along these domains.

## Methods

### Selection of study participants

Patients at five SNFs in the greater Philadelphia area who were admitted for short-term stays (sub-acute physical rehabilitation or skilled nursing needs after an acute hospital stay) were eligible for this study. The facilities purposively sampled for the study had high and low degree of physician focus on SNF practice (i.e., SNFist vs. non-SNFist facilities), were located in urban and suburban areas, and had a diverse population of patients. Consecutive patients were approached to participate if they were receiving post-acute rehabilitative care in the facility, had been in the facility for at least 5 but fewer than 10 days (to capture most patients based on the distribution of length of stay for post-acute care patients at US SNFs), and were English-speaking. Patients with severe cognitive impairment who were unable to provide informed consent were excluded [25]. Participants were asked for permission to contact their primary caregiver if they had one. For the purposes of this study, the person (e.g., family member or friend) who regularly assisted the patient with custodial needs, advised in medical decision making, or provided similar advice or assistance was considered the primary caregiver. If the caregiver was present at the patient’s bedside, they were recruited at the same time as the patient. Otherwise, the caregiver was contacted by telephone, screened to confirm their relationship with the patient, and recruited for participation in the study.

Of the 82 patients approached, 9 (11%) were ineligible for participation (8 could not provide informed consent, and 1 did not experience an acute hospital stay preceding their admission to the SNF) and 23 (28%) declined to participate. Of the 50 patients who participated, 31 (62%) identified a primary caregiver (i.e., family member, friend or neighbor providing physical care and emotional support during the patient’s illness, typically unpaid). Of those, 10 (32%) refused permission to contact the caregiver, and 15 (48%) caregivers declined to participate or could not be reached after three attempts. Of the 31 caregivers identified by the patients, only 6 (19%) participated in the study.

### Interviews

A semi-structured interview guide was developed by the authors (KR, NH, JK, JU), including two practicing geriatricians, and piloted with two SNF patients. The guide provided open-ended prompts for the interviewer to elicit the participants’ experiences and expectations of physician care during their SNF stay (Additional file 1). We used the term “prescribing provider” in the interview guide to include physicians, nurse practitioners, and physician assistants; however, nearly all respondents discussed physicians during the interviews. Therefore, we did not stratify the responses by type of prescribing clinician and could not generalize the findings to non-physician clinicians. Three undergraduate research assistants trained by the study team and two of the authors (KR, KF) conducted the interviews. The participants were interviewed in their room in the facility or another private location in the facility. If the primary caregiver identified by the patient was present at the time of patient’s interview and agreed to participate, they were interviewed together. Otherwise, the caregivers were interviewed over the phone. The same set of questions was used for patients and caregivers. The guide included questions about patient demographics, the preceding hospitalization, their expectations and experiences with physicians during the current SNF stay, and questions about their expectations of physician care after they leave the facility (e.g., primary care or follow up care). All patient participants were administered the Montreal Cognitive Assessment (MoCA) to measure cognitive impairment at the time of the interview. Caregiver participants were not asked to complete the MoCA.

### Coding

Interviews were digitally recorded, professionally transcribed (ADA Transcription, Westhampton, NJ), and cleaned to remove any information identifying individuals. The codebook was developed by four of the authors (KR, KF, BL, EG) trained in qualitative methods using grounded theory approach. Grounded theory approach is defined by inductive coding where researchers resist imposing preconceived assumptions to form a codebook and instead develop the codebook by reviewing data line by line until a new concept becomes apparent [26]. This is done iteratively until all key concepts are included in the codebook [26]. Each researcher independently reviewed a subset of the first twenty transcripts and identified potential structural codes to encapsulate interview questions and probes, as well as reoccurring concepts introduced by participants. The researchers then met to draft the first iteration of the study codebook, deciding on clear definitions and explicit inclusion/exclusion criteria for all codes. The initial codebook was then tested on five transcripts chosen at random, one of which was double-coded by the researchers who then came together to discuss how well the codes captured the data and to identify additional codes needed. After further refinement, the second iteration of the codebook was discussed by the study team to ensure that the codes were consistent with the research objectives. This discussion led to further modifications of the study codebook. The third and final iteration of the codebook was used to code all study transcripts, including both patient and caregiver interviews. While patients and caregivers represent two sources of data, consistency of information discussed and probed for in both sets of interviews allowed for one codebook to be applied to all data sources.

Coding was performed in an iterative manner over a five-month period while interviews were being conducted. This allowed the study team to add additional prompts to later interviews for more contextual information and to perform preliminary analysis of the first 20 interviews, identifying emerging themes to present to later participants as a form of member checking and to confirm theme saturation.

All coding was performed by two authors (BL, EG) with extensive training and experience in grounded theory qualitative analysis. To assess the level of coding agreement between the two coders, 10% of the transcripts were coded independently by both coders and a measure of agreement (the Cohen’s kappa coefficient) was calculated for each code within the double-coded transcripts. Any code with a kappa less than .60 required the two coders to review their coding and settle the discrepancies, and reach consensus on code definitions for subsequent coding. The mean inter-rater correlation coefficient (kappa) for the transcripts that were independently coded by two coders was 93%, and all of the text coded had kappas of 80% or higher, indicating high inter-rater reliability [27].

### Analysis

Using thematic analysis, two authors (KR, KF) independently analyzed all codes pertaining to SNF physicians across transcripts to identify recurring themes [26, 28]. The themes that emerged from early analyses were discussed by the study team and revised in iterative manner as more interviews were completed. After identifying major themes and sub-themes, the data was then stratified and queried by the degree of cognitive impairment (MoCA score) and facility characteristics (high vs. low degree of physician specialization in SNF practice) to determine whether the themes held true across these strata. To characterize patients by the degree of cognitive impairment at the time of the interview, we used the following categories: any impairment (MoCA < 26) vs. no impairment (MoCA 26 and above) [29]. To characterize the facilities by their degree of physician specialization in SNF practice, a weighted average of physician specialization in SNF-based practice was measured for each facility. To do so, the Medicare Provider Utilization and Payment Files [30] containing physician claims aggregated by service type were analyzed for all physicians who treated any patients in the study SNFs. First, the percentage of services provided by each physician to patients in SNFs was calculated relative to total services provided in any setting that year. Next, the percentages of SNF services for all physicians within each facility were weighted by the proportion of patients each physician treated in the facility and averaged across all physicians who treated patients in that facility.

A detailed audit trail was maintained throughout the study, including interview notes and debriefing, codebook development, resolution of coding inconsistencies, and analyses [31]. After each of the last nine interviews, two authors (KF, KR) reviewed the major themes and select subthemes with the participant immediately following the conclusion of their interview. The participants were asked to indicate whether they felt the statements captured their experiences with physicians during the SNF stay. The participants’ affirmation of these themes allowed the authors to conclude interviewing due to thematic saturation, when additional interviews revealed no new themes [26].

Coding and analyses were performed using NVivo, Version 12 (Melbourne, Australia). The study was reviewed and approved by the Institutional Review Board of the University of Pennsylvania.

## Results

### Sample characteristics

A total of 50 SNF patients and 6 caregivers participated in the study. Half (48%) of participants were female; 20% were younger than 65 years old, 26% were between 65 to 74 years of age, 36% were between 75 to 84 years of age, and 18% were over 85 years old at the time of the interview. Of the sample, 52% were white, 38% black, and 10% other race(s). Most patients (92%) completed high school or higher education. At the time of the interview, 34% of patients interviewed had no detectable cognitive impairment, 43% of patients had a mild degree of cognitive impairment, and 23% had moderate to severe cognitive impairment (Table 1).

**Table 1:** Study Sample Characteristics (*N* = 50)^a^

Characteristic	Frequency	Percentage
Gender		
Male	26	52%
Female	24	48%
Age		
< 65	10	20%
65–74	13	26%
75–84	18	36%
≥85	9	18%
Race		
White	26	52%
Black	19	38%
Other	5	10%
Education		
Some high school or less	4	8%
High school diploma	18	36%
Some college	7	14%
College degree	12	24%
Graduate degree	9	18%
MoCA score (*n* = 44)		
High (26–30)	15	34%
Intermediate (20–25)	19	43%
Low (Less than 20)	10	23%

^a^Patients only. We did not collect demographic information or perform MoCA assessments of caregiver participants

### Major recurrent themes

Table 2 shows the three major recurrent themes and their sub-themes that describe the participants’ expectations and experiences of physician care in SNFs. Those major themes were: (1) participants had poor awareness of the physician(s) in charge of their care at the facility (awareness was defined as knowledge of identifying characteristics [e.g., name, appearance, contact information] or professional activities [medical services or expertise provided, or typical role in relation to other staff such as nurses, hospital physicians, external consultants]); (2) they were dissatisfied with the frequency and quality of communication with the physician(s); and (3) participants valued the perception of receiving individualized care from their physician(s). The themes and select sub-themes are described in detail below. All sub-themes and their representative quotes are presented in Table 2.

**Table 2:** Participant Perceptions of Their Experiences and Expectations of Physician(s) Care in Skilled Nursing Facilities

Theme	Sub-theme	Representative quotes
Poor awareness of the physician(s) in charge of their care at the skilled nursing facility	Inability to name or otherwise identify the physician(s) overseeing their care	“I’ve never really talked to him...I didn’t even know he was a doctor.” – Female, 70’s-80’s years old
	Expectation that external providers (i.e. their surgeons, hospitalist, or primary care provider) would be directing their post-acute care at the skilled nursing facility	“From what I’ve been told there’s a separate physician here. It’s not my surgeon, which I’m not crazy about. I mean, he gave me the surgery, he should be in here to check on meat least once or twice during the 10 days I’m here. But he hasn’t been. It’s been a different doctor who was only here one time.” - Female, 60’s-70’s year old
	Confusion about the distinct roles of physicians, nurse practitioners, nurses, and nursing assistants on the care team	“It’s very hard to tell who’s a doctor. That person, I guess, he’s a doctor, it happens every once in a while, a guy comes in with a suit and tie on and he doesn’t really introduce himself. He just sits down and we start talking about things. I wish they would announce themselves - exactly who they are.” – Male, 60’s-70’s years old
	Distress associated with the lack of understanding about the physician(s) coordinating their care	“I don’t know [if there is a separate doctor or nurse practitioner who coordinates my care in this facility]. I don’t know. I feel kind of stupid not knowing.” - Female, 70’s-80’s years old
Frequency and quality of communication with the physician did not meet participants’ expectations	Frequency of communication did not meet expectations	“I mean, if I need a question they always tell me to ask the nurse. And Dr. [last name], I only saw him once and I probably won’t see him again this week.” – Male, 50’s-60’s years old
	Quality of communication did not meet expectations: perceived to be rushed, superficial, insufficient to learn patient preferences for care	“I think that [physicians] can stay on top of stuff a little bit more. I know they have a lot of patients here and I know they’re really busy, but I just feel like I’m like their supervisor and I’m keeping on top of them and making sure they do their job. And I don’t think I should be doing that. I don’t want to have to be burdened with that mindset that I need to stay on top of these people in order for them to do what I need to have done.” – Female, 60’s-70’s years old
	Caregivers expected more frequent and detailed communication with the physician	“I thought [the physician] should have had a little more [communication] with me when I asked him a question. I told him, ‘[My father] is an [80’s] year-old man. If you have a question, you need to call me.’”– Female, 30’s-40’s (caregiver)
Participants valued care that was perceived to be individualized to their needs by the physician(s) in the facility	Perception of physician(s) being dismissive of the patients’ symptoms	“I didn’t care for the doctor there because she acted like I didn’t know my own body and I didn’t know what I was talking about. And I know how much insulin I need, because I give it to myself, because I’ve been a diabetic for [many] years, which is a long time.” – Female, 70’s-80’s years old
	Appreciation of specialized care to meet individual needs	“And [the physician] is very understanding of my father and how he thinks. And thinking so much about my father and then specifically understanding of his needs, like the [diet] element, things like that. Some people really shake that off like it’s not important – he has to eat – but for a man who spent his life that way, it is important.” – Female, [declined to provide age] (caregiver)
	Patients felt they were a burden due to their medical complexity or custodial needs	“You know one thing I think about this place – they knew that I was [medically complex and high needs] – why did they accept me? Don’t accept people because you need patients. You accept them because you wanna help them.” – Female, 70’s-80’s years old

### Poor awareness of physician(s) in charge of the Patient’s Care at the Facility

Patients and their caregivers had poor knowledge of their physician(s) at the SNF. Nearly all patients interviewed were either unable to name or misidentified the physician in charge of their care at the facility. For example, a prevalent misconception among patients was that the attending physician who treated them during the hospital stay that preceded the SNF stay was directing their care and prescribing their medications. As a result, many patients expressed disappointment when they were unable to see their hospital attending physician (or clinician team) after admission to the SNF. For example, a patient discussed her disappointment that her operating surgeon did not have a continued role in her care at the SNF,

“From what I’ve been told there’s a separate physician here. It’s not my surgeon, which I’m not crazy about. I mean, he gave me the surgery, he should be in here to check on me at least once or twice during the 10 days I’m here. But he hasn’t been. It’s been a different doctor who was only here one time.”

- (Female, 60’s-70’s years old)*.*

Active involvement in their post-acute care (including in-person visits, frequent communication, medication management) by the attending physician in charge of their hospital stay was an expectation of patients admitted to the SNFs for post-acute care, and patients expressed disappointment when this expectation was not met.Participants were also frequently confused about the roles of different healthcare providers in the SNF. Other than the misconception that hospital attending physicians were in charge of their care at the SNF, participants did not have any expectations of physician care at the facility. Patients described the intake process at the facility that typically included interactions with different members of the clinical team, but reported confusion about individual clinicians’ roles. For example:

“I'm not sure who makes decisions about my medications at [this facility]. I know there is a plan, and they asked me different things, if I need this or that or the other, but I don't know who is actually–whether it's the nurse, whether it's a supervising physician. I assume it's a supervising physician, but I don't know.”

- (Male, 70’s-80’s years old).

As a result of these misconceptions and confusion, participants reported difficulties accessing their physicians to change their medications, address new symptoms, or discuss the plan of care. Many patients described feeling disempowered to take an active role in their care. Patients were uncomfortable with their lack of knowledge of the physicians in charge of their care and stated that it made them uneasy. For example, one participant said,

“I don’t know [if there is a separate doctor or nurse practitioner who coordinates my care in this facility]. I don’t know. I feel kind of stupid not knowing.”

- (Female, 70’s-80’s years old).

### Frequency and quality of communication

Participants reported dissatisfaction with the frequency and quality of communication with the physician(s) at the SNFs. Most patients saw the physician no more than once a week during their stay but expected more frequent communication with the physician(s). Likewise, the caregivers interviewed also reported that they felt the frequency of communication was inadequate, and they were dissatisfied with their access to the physician(s). One patient discussed frustrations with unclear expectations of the frequency of interaction with their SNF physicians, stating the following:

“I think the case managers and the hospital need to make sure the patients understand that…you're not gonna see a doctor every week.” - (Female, 60’s-70’s years old)*.*

Participants also reported that the quality of communication did not meet their expectations. They perceived physicians’ interactions with them as rushed, superficial, and insufficient to learn the patient as an individual and understand their preferences for care. Participants also perceived that physicians do not communicate adequately with external physicians in other facilities (e.g., hospital, primary care physicians), leading to disorganized and delayed care delivery. For example, participants assumed that inadequate communication with the discharging hospital’s physicians resulted in a lack of staff preparedness for intake upon their arrival to the SNF. Patients also perceive inadequate communication between the physicians and other SNF staff. They observed a lack of physicians responsible for care coordination and supervision of the care delivered to them by direct care staff, a role typically attributed to the physician in the hospital and clinic. One patient reported that he had to coordinate his own care, while another felt that she needed to act as the “supervisor” to SNF staff in order to receive care that she felt was adequate:

“I think that [physician] can stay on top of stuff a little bit more. I know they have a lot of patients here and I know they’re really busy, but I just feel like I’m like their supervisor and I’m keeping on top of them and making sure they do their job. And I don’t think I should be doing that. I don’t want to have to be burdened with that mindset that I need to stay on top of these people in order for them to do what I need to have done.” – (Female, 60’s-70’s years old)

Participants who experienced physician communication that did not meet their expectations felt that their physician(s) in the SNF did not know them or their preferences for care. Some caregivers expected more frequent and detailed communication with the physicians(s) in the facility especially because their loved one was in the facility for a short-term stay and the facility staff did not know the patient. For example, one caregiver said:

"I thought [the physician] should have had a little more [communication] with me when I asked him a question. I told him, '[My father] is an [80’s] year-old man. If you have a question, you need to call me.'" – (Female, 30’s-40’s years old, caregiver)

Participants attributed other perceived deficiencies in their care at the facility to the infrequent and superficial communication with the SNF physician(s). Patients and caregivers experienced distress and felt neglected if they experienced communication with the physicians that did not meet their expectations. One patient felt that the SNF physician “doesn’t give a toot” about his/her patients.

### Perceptions of individualized care from the physician(s) associated with more positive SNF experience

Patients valued several aspects of the care they received from the physician(s) in the SNFs. One major recurrent theme was that participants who perceived that their care was individualized to their needs had more positive opinion of their physician(s). In contrast, patients who perceived that their physician(s) did not take time or make effort to understand their individual needs were likely to have a negative opinion of the physician’s care. For example, some patients perceived that their physician did not take their concerns seriously:

"I didn’t care for the doctor there because she acted like I didn’t know my own body and I didn’t know what I was talking about. And I know how much insulin I need, because I give it to myself, because I’ve been a diabetic for [many] years, which is a long time." – (Female, 70’s-80’s years old)

Patients also frequently expressed this sub-theme about pain management. When patients discussed their pain management, it was often to express concerns that the SNF physician(s) were dismissive of their symptoms, with one patient expressing that her experience made her feel like “some kind of drug addict”. In contrast, many participants described positive experiences with individualized care, including one patient’s caregiver who appreciated their physician’s awareness of special dietary needs:

"And [the physician] is very understanding of my father and how he thinks. And thinking so much about my father and then specifically understanding of his needs, like the [diet] element, things like that. Some people really shake that off like it’s not important – he has to eat – but for a man who spent his life that way, it is important." – (Female, [declined to provide age] (caregiver))*.*

Many patients stated that their overall experience with their physician in the facility was positive, even when they had discussed specific, negative aspects of their care. Overall, patients were satisfied with physician care, often stating that their physician(s) were “nice” and that the care was “good”. Many patients felt that they required very complex and intensive care due to their medical needs and physical limitations. On one hand, patients expressed appreciation when those needs were recognized and met. On the other hand, some patients reported feeling like they were a burden to the facility:

"You know one thing I think about this place – they knew that I was [medically complex and high needs] – why did they accept me? Don’t accept people because you need patients. You accept them because you wanna help them." – (Female, 70’s-80’s years old)

### Stratified analyses

Participant perceptions of the physician(s) care in SNF were generally consistent for patient with vs. without cognitive impairment. Patients who were less cognitively impaired based on their MoCA score during the interview expressed confusion and poor knowledge of their physician who was directing their care in the facility, as did patients who were more impaired (based on their MoCA score). One notable distinction was that patients who were less cognitively impaired expressed concern about the lack of knowledge of the physician(s) at the facility, whereas more cognitively impaired patients were less concerned about their lack of awareness of the physician(s) at the facility.

Participants who were more cognitively impaired were more likely to confuse the different roles of physicians, nurse practitioners, nurses, and other SNF personnel when discussing their care teams. In contrast, participants who were not detected to have cognitive impairment based on MoCA performance were generally able to accurately describe the differences in the scope of practice of physicians vs. other staff. However, similar to cognitively impairment patients, many cognitively intact patients were unable to identify their physician(s) at the facility by name. Patients without cognitive impairment were more likely to be aware of this gap in their knowledge about their care team and to express concern about that.

Patients in SNFs with physicians who focused their practice in SNFs (i.e., SNFists) were more likely to report positive experiences with physician care as well as with their overall experience at the facility compared to the patients in SNFs where physicians did not focus their practice exclusively on SNF-based care. However, we did not detect differences in the major themes across participant perceptions between facilities with high vs. low degree of physician ‘specialization’ in SNF practice.

## Discussion and implications

Patients and their caregivers have a limited understanding of staffing and care processes in SNFs. Misinformation about the availability and role of physicians at the facilities leads to unmet expectations and dissatisfaction with overall care experience. Patients across a wide range of cognitive function reported concerns regarding aspects of physician care in the facilities, including infrequent communication, inadequate treatment of symptoms, and poor rapport with the physicians. Patients and caregivers who perceived that their physician understood their individual needs and aimed to provide care to meet those needs were more likely to report a positive experience with the physician.

These findings are consistent with the expectations of the Medical Staff Involvement Model in some areas and deviate from the conceptual model in others. First, patients and caregivers have expectations of their SNF physicians along the domains of communication and care coordination (i.e., individualized needs), consistent with the Medical Staff Involvement Model. Second, patients in SNFs with physicians who focused their practice in SNFs (i.e., SNFists) were more likely to report positive experiences with physician care as well as with their overall experience at the facility compared to the patients in SNFs where physicians did not focus their practice on SNF-based care. This is also consistent with the Medical Staff Involvement Model, which conceptualizes job commitment of physicians as a key dimension of facility quality. On other hand, contrary to our expectations based on the Medical Staff Involvement Model, we did not observe differences in the major themes between patients in SNF facilities stratified based on physician staffing patterns. Considering substantial growth in the proportion of SNF care provided by SNFists (compared to non-SNFists) in the US, more research in this area is urgently needed to identify drivers of positive patient and caregiver perceptions of SNFs with more specialized physicians (vs. more generalist physicians).

Prior studies of physician practice in SNFs focused primarily on care processes and quality at the end-of-life. In 2000, a large national study surveyed family members and other informants about end of life care for 1578 decedents reported concerns with physician communication and perception of lack of respect toward long-term care residents [20]. In qualitative follow-up interviews to that study, 54 family members of patients who died in a nursing home reported low expectations of physician-level care overall and specifically a lack of physicians to address the needs of dying patients [18]. Poor quality of communication with physicians and the need to advocate on their loved ones’ behalf to assess and treat symptoms at the end of life contributed to the caregiver burden [19]. In a different study, among patients with dementia transferred to SNFs after a hospitalization, poor and incomplete communication between external physicians and SNF nurses was associated with poor patient outcomes and increased caregiver stress during the transition [32]. A recent study using 2012 to 2014 Medicare billing data for short-stay patients in SNFs found that while 71.5% of patients saw a physician within 4 days after admission to the facility, there was considerable variation in the timing of the first visit, and 10.4% of patients did not have a visit billed to Medicare during their SNF stay [33]. The findings of this study are consistent with the earlier qualitative reports and the more recent quantitative findings of inadequate patient access to physician care in SNFs.

We know that strong physician-patient alliance is associated with improved outcomes across a range of medical conditions [11–13]. Awareness and understanding of patient expectations is key to building physician-patient alliance and improving patient experience and outcomes of care. Current measures of SNF quality such as Medicare’s Nursing Home Compare ratings do not include patient-reported measures, such as their perceptions of physician care [34]. The CAHPS Nursing Home Surveys developed by AHRQ [35] include questions about select aspects of the patient’s experience at the facility that relate to physician care, however, these surveys are used internally by SNFs for quality improvement activities and their results are not currently available to the public. The findings of this study offer confirmatory evidence that patients and caregivers perceive physician care to be important to their overall satisfaction with the care they receive in SNFs. Furthermore, patients with varying degree of cognitive impairment reported similar concerns about specific aspects of their experience with the physicians at the facilities, including awareness of the physician team, frequency and quality of communication, and perceptions related to the physician(s) effort to provide individualized care. Assessment of cognition using MoCA at the time of the interview, albeit an imperfect measure of cognitive function, nevertheless underscores the fact that misconceptions about physicians in SNFs are not limited to patients with cognitive impairment. In fact, we found that patients with greater cognitive impairment were less concerned about their lack of knowledge of physicians in charge of their care at the facility, compared to unimpaired patients who were more distressed.

One strategy to improve access to physician level care in SNFs is to use physicians (or advanced practitioners) who focus their practice in this setting. The prevalence of physicians who specialize in SNF based practice (colloquially known as “SNFists”) increased by 34% between 2012 and 2015 [7]. There is evidence that patients under the care of SNFists have better outcomes. In a national study of facilities with both types of physicians, patients under the care of SNFists had 1.5% lower rehospitalization rate and 0.8% higher rate of successful discharge to community [10]. Little is known about patient and caregiver experiences and perceptions of SNFist care. We found that patients in facilities with SNFists had generally more positive experiences with their care, but did not detect specific differences in practice that may explain higher satisfaction. One possible explanation is that our study did not account for the number of facilities each physician visits. For example, a SNFist who treats patients across many different facilities may have unique expertise in this patient population but might not have enough presence in any single facility to change the perceptions of patient and caregivers. Future research should evaluate the role of patient panel size and number of facilities in patient outcomes of SNFist care.

This study has several limitations. First, we recruited participants from Philadelphia-area SNFs and our findings may not be generalizable to other areas. However, facilities that participated were diverse across suburban and urban locations, patient demographics, and the physician(s)’ focus on SNF-based practice. Second, only 40% of the patients in our sample had a primary caregiver based on our a priori definition for this study and only a fraction of those caregivers agreed to participate in an interview. This may be due to the fact that the short-term stay patients included in our sample are higher functioning and more independent than the overall nursing home population that includes more dependent long-term care patients. The small number of caregivers precluded evaluation of any differences between participants who could speak for themselves vs. participants who relied on a caregiver to speak for them because of their severe degree of cognitive impairment. Nevertheless, our sample included patients who were admitted to the SNF with long-term plans to transition to custodial nursing home care after their skilled nursing needs were met, was demographically diverse and included participants with varying degrees of cognitive impairment. Third, we were unable to triangulate the participants’ responses with the medical record documentation or observations of patient interactions with the physicians at the facilities. Thus, our findings are limited to the perceptions of physician care in SNFs and may not reflect actual practices. Furthermore, we did not characterize patients’ or caregivers’ previous experiences with medical professionals, which may inform their expectations and perceived experiences during the current SNF stay. Future studies should compare patient and caregiver experiences with physician care in SNFs to their experience with physician care in other settings (e.g., acute rehabilitation facilities).

Our findings have several important implications for SNF practice and policy. First, discharging facilities and accepting SNFs should set expectations about physician practices in the facility that closely resemble the reality. These expectations could be relayed to the discharging facilities so that patients are better prepared for the experience once they are in the SNF. Second, public reporting initiatives such as Nursing Home Compare may consider incorporating measures of physician care quality in global facility rating used for public reporting. Patients and caregivers clearly care about this aspect of SNF care. Such measures could be strengthened by triangulating the information reported by patients and caregivers with perceptions of SNF staff, administrators, and policy makers. Third, policies and practices related to regulatory practices and reimbursement should aim to improve access of SNF patients to physicians. There is a relative dearth of policy initiatives that aim to improve patient access to physicians in SNFs. This is particularly important in the context of healthcare delivery reforms spurned on by the COVID-19 pandemic, and may improve patient and caregiver satisfaction with post-acute care in SNFs.

## Conclusions

Patients and caregivers perceive the physician care they receive as not meeting their expectations. In particular, after admission from the hospital to a SNF, patients expect follow up from the physician in charge of their care in the hospital as well as more frequent and in-depth communication with the new physicians in charge of their care in the SNF. Our findings highlight persistent deficits in access to physicians in the SNFs, from the perspective of patients recently discharged from the hospital to short stay in SNFs and their caregivers. Given the high risk of adverse outcomes in this population, efforts to address these concerns are warranted.

## Supplementary Information

**Additional file 1.** Appendix.docx. SNF Caregiver & Patient Expectations & Experiences Interview Script. Clean copy of the interview script.

## Data Availability

The datasets generated and analysed during the current study are not publicly available due to confidentiality concerns given the small sample size and qualitative nature of the data, but may be made available in de-identified form from the corresponding author on reasonable request with corresponding ethics board review and approval.

## Abbreviations

MoCA: Montreal Cognitive Assessment
SNF: Skilled nursing facility
SNFist: Skilled nursing facility ‘specialist’ (physician or advanced practitioner such as nurse practitioner or physician assistant with a practice focused on SNF-based care)
