# Supplementary material for: Expectations and experiences with physician care among patients receiving post-acute care in US skilled nursing facilities
Source: BMC Geriatr. 2020 Nov 10;20:463. doi: 10.1186/s12877-020-01869-1 (PMC7653446; doi:10.1186/s12877-020-01869-1)
Supplement: Supplementary file 1 — Additional file 1. Appendix.docx. SNF Caregiver & Patient Expectations & Experiences Interview Script. Clean copy of the interview script. [file 12877_2020_1869_MOESM1_ESM.docx]

**Appendix**

**SNF Caregiver & Patient Expectations & Experiences Interview Script**

Introduction

Hello, my name is _______. I am a researcher at Penn Medicine and we are conducting interviews to learn more about the care of patients in skilled nursing facilities. The purpose of this interview is to better understand the experiences and expectations of patients recently transitioned from hospitals to skilled nursing facilities. We are specifically interested in your expectations and experiences around prescribing clinician care. Prescribing clinicians means doctors or nurse practitioners who diagnose and treat your condition, sometimes with the help of specialist consultants or clinician-trainees. Prescribing clinician is someone ultimately responsible for your medical care.

When we begin the interview, I will start a recorder in order to document the interview. These recordings are used in order to ensure that there are no mistakes in my notes. Your name and information will not be connected with any reports and we will keep your information confidential. We will not share your comments with other staff or administrators. To remind you, your participation in this interview is completely voluntary and may be stopped at any time without any repercussions or impact on your healthcare.

Do you have any questions before we begin? [start recording]

This interview is being conducted by _______________[interview name] on __________[date] with Participant Number ___________.

Demographic questions

1. What is your age?
2. What is your gender?
3. What is the highest level of education you have completed?
4. What is your race or ethnicity?
5. What injury or illness brought you to this rehab facility?
6. How long were you in this rehab facility?

*Ok. Now I’d like you to think about your primary care (or main) doctor or nurse practitioner taking care of you BEFORE you were hospitalized…*

Open-ended Questions — For patients without dementia and patients with mild to moderate dementia

1. Please describe your relationship with your/your loved one’s primary care doctor or nurse practitioner before this hospitalization.
2. *Prompt*: Is there a “regular” doctor you/your loved one saw regularly?
3. *Prompt:* Was this person also seeing you/your loved one during the hospital stay?
4. *Prompt:* How frequently did you/your loved one see this clinician? Monthly, weekly, etc.
5. *Prompt:* Do you feel like the clinician knew your/your loved one’s medical condition, preferences for treatment?
6. *Prompt:* Can you describe how you know that the clinician knew you/didn’t know you? Can you think of an example of your regular doctor taking care of you (for example a health problem you went to see them for). How did that go?
7. Please describe the experiences you/your loved one had with prescribing clinicians (i.e. the physician, nurse practitioner, or physician’s assistant who prescribes you/your loved one medication) during this hospital stay, including how many clinicians you/your loved one saw, how often you/your loved one saw clinicians, and how these clinicians treated you/your loved one. [pause for an answer] What were your expectations regarding prescribing clinician care in the hospital and in what ways were these expectations met, failed, or exceeded?
8. *Prompt:* What was the role of these clinicians as you understood it?
9. *Prompt:* Were you satisfied with their care?
10. Prompt: For example, how often did you expect to see a clinician? Daily, every few days?
11. Please describe how you came to the decision to move yourself/your loved one from the hospital to a skilled nursing facility (i.e. the nursing home in which you are currently being cared for) and please speak about everyone who was involved in this decision making process and describe their role in it.
12. How would you characterize your/your loved one’s transition from the hospital to the skilled nursing facility? Additionally, what were your expectations regarding this transition and in what ways were these expectations met, failed, or exceeded?
    - - 1. *Prompt:* Was it organized? Were the different roles of clinicians during this process made clear to you?
        2. *Prompt:* If disorganized, then ask about how medical information, preferences for treatment, etc were transferred across settings.

*OK Now I’d like you to think of your care in this rehab facility…*

1. Do you know who makes decisions about yours/your loved’s ones medications and treatment in this facility?
   - - 1. Is this clinician a different clinician or the same person who was taking care of you/your loved one before coming to this facility?
       2. How many different clinicians are taking care of you during your/your loved one’s stay in this facility?
       3. *Prompt:* If there is more than one clinician, do you think the clinicians communicate effectively with each other regarding your/your loved one’s care.
       4. *Prompt:* Can you describe an example of how clinicians communicated about your care?
2. How often does your/your loved one’s prescribing clinician (i.e. the physician, nurse practitioner, or physician’s assistant who prescribes you/your loved one medication) in this facility communicate with you/your loved one? (i.e. daily, several times per week, weekly)
   *To the caregiver:* How often does your loved one’s prescribing clinician communicate with you? (i.e. daily, several times per week, weekly)
   - - 1. *Prompt:* Do you think this is an appropriate amount of communication?
       2. *Prompt:* What were your expectations regarding communication with prescribing clinicians in this facility and have they been met, failed, or exceeded?
       3. *Prompt*: Was there ever a time you needed to reach the prescribing clinician and could not?
3. How well do you feel the nursing home’s prescribing clinician (i.e. the physician, nurse practitioner, or physician’s assistant who prescribes you/your loved one medication) knows you/your loved one?
   - - 1. *Prompt*: Do the clinicians know your/your loved one’s medical history and conditions?
       2. *Prompt:* Do the clinicians know your/your loved one’s preferences for treatment?
4. What role does your/your loved one’s prescribing clinician (i.e. the physician, nurse practitioner, or physician’s assistant who prescribes you/your loved one medication) play in their care in this facility?
   - - 1. *Prompt*: How does the clinician work with the rest of the nursing home staff? Do you think that the clinicians communicates effectively with the nursing home staff?
5. How would you characterize the quality of care that you/your loved one is receiving from their prescribing clinician (i.e. the physician, nurse practitioner, or physician’s assistant who prescribes you/your loved one medication)? Please include specific details about the care you/they are receiving.
   - - 1. *Prompt*: How does your/your loved one’s prescribing clinician (i.e. the physician, nurse practitioner, or physician’s assistant who prescribes you/your loved one medication) treat you/them?
       2. *Prompt:* Does the prescribing clinician listen to you/your loved one? Are they an effective or compassionate communicator?
       3. *Prompt:* Do they treat you/your loved one with respect and give you/them proper attention?
6. In what ways have your experiences with your/your loved one’s prescribing clinician(s) in this facility met, failed to meet, or exceeded your expectations for your/your loved one’s care while in a skilled nursing facility?
   - - 1. *Prompt:* Do you have any other comments about your/your loved one’s clinician (i.e. the physician, nurse practitioner, or physician’s assistant who prescribes you/your loved one medication)?

*Now I’d like you to think ahead about your discharge from the rehab facility….*

1. What is your expectation about who will be your/your loved one’s primary clinician (the doctor or nurse practitioner that you see regularly for care) after you leave the nursing home. Do you have any concerns, preferences, or expectations regarding that?

*Now, I have to ask you a question that I need to ask everyone. Do you have memory problems? Have you ever been diagnosed with a cognitive impairment or dementia?*

[conduct MoCA — see MoCA guidelines]

For patients with mild to moderate dementia (i.e. if the patient reports being diagnosed with dementia or patients)

1. Do you think the clinician knows how to effectively communicate with persons with dementia? Do you think they understand the needs of persons with dementia?
2. Do you know if your/your loved one’s clinician has experience caring for persons with dementia?
